# On the Arrest of Typhus Fever by Quinine

**Published:** 1852-11

**Authors:** Robert Dundas

**Affiliations:** Physician to the Northern Hospital, Liverpool


ART. VI.—*On the arrest of Typhus Fever by Quinine.* By Dr. ROBERT DUNDAS, Physician to the Northern Hospital, Liverpool. A review thereof by SCHRAPENKUTTEL SMELFUNGUS, M. D.

Doctor Smelfungus has a long nose wherewith he "scents the battle afar off." He not only scents the battle, but his olfactories dilate readily to malaria and marsh miasm. Surmounting this nose of Dr. Smelfungus, is a head, said by some to be not so long as his nose. But that head, such as it is, has been lately much pothered by the question of the existence, or non-existence, of miasmatic emanations as a blood poison.

Dr. Flint intimates that it is an open question. Dr. Robert Dundas, of the Northern Hospital, Liverpool, Eng., says, point blank, that "ague and remittent fever do not originate in malaria, or marsh miasm."

Dr. Hamilton, *et multi alii*, says, that malaria does cause, not only intermittent and zymotic diseases, but also Asiatic cholera.

Dr. Smelfungus says, with an emphasis, "Amen," and fixing his eyes upon his little collection of medical authorities, (his best friends and solace of many an hour otherwise lonely,) declaims:

"If we are to throw away this time-honored doctrine of malarial poison, with it must go all measures of sanitary reform. We may with impunity build our dwellings on the easterly side of miasmatic swamps — a cottage by the border of the stagnant Bayou is as healthful as a home upon the breezy hillside. Dickens has written in vain his wonderful descriptions of the fever haunts of London, and 'Tom-all-alone's' may remain in chancery till the crack of doom without detriment to the public health — all notions of ventilation, and drainage, and cleanliness are obsolete — we may erect our temples of Cloacina beneath our bedroom windows, and inhale the odors of their vaults through the long summer nights, as we would 'the scent of orange bowers' — we may wallow in all manner of uncleanliness, and revel in the filth of Asiatic cities, without the danger of their plagues."

"Now," says Dr. Smelfungus, growing milder, "I am extremely anxious that Dr. Dundas should make good his second proposition, which I find in the last number of 'Braithwaite,' viz., that intermittent, remittent, and continued fevers are but different varieties of the same disease, and all equally curable by the same means, viz., *quinine*, (glorious old *protege* of the Society of Jesus,) in doses large enough, viz., 10 grs. every two hours, continued for some thirty-six hours. I have faith in that latter idea. I have long been regardless of the existence of fever, when about to give a *large* dose of that drug. *I know quinine*, and there is good sense, and philosophy in supposing that given in these doses it would do what Dr. Pitcaim, and Southwood Smith, and Tweedie, and all the rest of the 'old fogies' said was impossible, *id est*, cut short a typhus fever. But," (growing warm again) "why in the name of all that is rational, should he block the way to trial of his mode of cure by connecting with it this *nouveau né* hobby of no malaria? Dr. Dundas! 'I know a vale,' where let me place you for a few hours at the rising of the sun and the going down of the same — you may take a nap if you please — all the better — and you shall be nicely covered up with a little fog blanket, which smells as if it had been slept in before — but you shall come out of that valley, with such evidence of the existence of malaria, as shall make you *shake* in your shoes!"

Dr. Smelfungus grows contemplative, and with a long drawn sigh of pity for human frailty and short-sightedness, takes a chew of Nicotiana Tabacum. It is not his first, for already upon the floor is a miniature diagram of the whole chain of lakes. This fixes his eye, and he gently comments on the different localities — "There is Chicago, and Racine, and Milwaukee, all having the four essentials of malaria, air, moisture, heat, and vegetable decomposition, all fever localities. And there is Mackinaw — high, breezy Mackinaw, where people have to move away to die; and there are Toledo, and Sandusky, and the deadly Maumee valley, where the arrival of the cholera predisposition was but the touching of the torch to the full magazine. There, too, is Cleveland, on its noble bluff, with sandy soil, and open parks, and freedom from disease. There, again, is Buffalo — 326 deaths from cholera there this healthy year, among those canals and mud-puddles, while the south-west wind sweeps up from Hamburg swamp with death upon its wings.

Finally, there is Rochester — city of Universities, and Athenæums, and Eclectic Medical Colleges! with its truculent board of health, which valorously tears down all nostrum handbills, and returns to a prominent physician his respectful communication, wherein he tells them that right in the heart of their city — in the dry bed of their river, is the source, and fount of that vial of wrath which has been poured out without measure on their devoted town."

Dr. Smelfungus is satisfied with himself. He takes another chew, and thus doth cogitate: "Why is it that Dr. Dundas doubts, or denies the old theory of malarial poison? He has lived in Brazil — he has seen tropical diseases. He says that continued fevers do occur in hot climates, and that they not uncommonly mutate to the remittent form. Well, what of it? The old idea of a special animal poison, in the causation of continued fever, is tolerably well exploded. If all these forms of fever are the same, (and for aught I care you may add yellow fever to the list,) why may not they have the same cause, and where is the wonder in their changing forms?

"Remittent fevers assume a typhoid type. Dr. Fenner says that bilious remittent in the great heats, and close air of a city, may become yellow fever. Why, then, may not, under favorable circumstances, a continued fever become remittent, and if it does, what bearing has this on the great question whether marsh miasm causes intermittent and remittent. Marsh miasm is an unfortunate term. Dr. Caldwell told us twenty years ago, that a marsh was not necessary to the production of malaria, and proved it, too, in a certain prize essay. But malaria — is it not possible that Dr. Dundas has been wool gathering among researches as to what malaria is, till disappointed at its intangible nature, he has grown to deny its very existence. If so he has grasped at a fog, and found nothing but a shut and empty hand. Laucisi long ago upset the old animalcular theory, which supposed that myriads of little monads issued from the marshes to insinuate themselves with disgusting familiarity into our mouths, and noses, and our very pores. But he did not tell us what the thing is. Others have tried to do so, and sulphuretted hydrogen, and carburetted hydrogen, and phosphoretted hydrogen, nitrous acid gas, and every other gas, and conceivable combination of gases, have had their earnest advocates, but in vain.

"Doctor Smelfungus opines, that it is (in very original phrase) a subtle essence, which, generated by the action of solar rays upon decomposing vegetable matter in a moist state, produces our ordinary autumnal fevers — that when acted upon by *long continued tropical heat*, it causes the deadly congestive and yellow fevers, but that when produced by artificial heat, in winter, it (conjoined with such other causes as unite in a crowded ship load of emigrants) gives birth to the continued forms of fever. Not only this — it may, under favorable predisposing causes, produce dysentery, diarrhœa, and cholera.

"Furthermore, Dr. Smelfungus saith not, save this, that his theory above announced is a very convenient one, and harmonizes all discrepancies. If you ask *why* quinine cures continued fever, he tells you because it cures remittent; if you ask why it cures remittent, he cunningly shutteth one eye, and propounds the following *hard question* —why does a chimera, ruminating in a vacuum, devour second intentions?"

Dr. Smelfungus looks at his bulls-eye watch, and finds himself among "the wee sma' hours ayont the twal'." He turns with a sigh to his bachelor bed, and breathes a prayer for the speedy coming of that day, when a Mrs. Doctor Schrapenkuttel Smelfungus shall be a blessing to his basket and his store, and a warming-pan to his couch o' these chill nights. *Festina diem!*

